# The Inventory of Personality Organization: A valid instrument to detect the severity of personality dysfunction

**DOI:** 10.3389/fpsyt.2022.995726

**Published:** 2022-11-10

**Authors:** Zsolt Unoka, Krisztina Csáky-Pallavicini, Zsolt Horváth, Zsolt Demetrovics, Aniko Maraz

**Affiliations:** ^1^Department of Psychiatry and Psychotherapy, Semmelweis University, Budapest, Hungary; ^2^Doctoral School of Psychology, Eötvös Loránd University (ELTE), Budapest, Hungary; ^3^Institute of Psychology, Eötvös Loránd University (ELTE), Budapest, Hungary; ^4^Centre of Excellence in Responsible Gaming, University of Gibraltar, Gibraltar, Spain; ^5^Institute of Psychology, Humboldt-Universität zu Berlin, Berlin, Germany

**Keywords:** personality functioning, personality impairment, personality disorganization, personality disorders, assessment, Inventory of Personality Organization

## Abstract

**Background and aims:**

In the eleventh revision of the International Classification of Diseases (ICD-11), the severity of personality dysfunction became the central dimension of personality disorder’s (PDs) definition, besides the trait domain qualifiers. Personality functioning, also known as personality organization (PO), is becoming an increasingly important concept in administering, predicting, and measuring severity and nature of personality disturbance. Otto Kernberg and his team developed several tools to measure personality impairment. The Inventory of Personality Organization (IPO) is a self-report rating scale for the measurement of PO. Aim of this study was to identify severity groups according to the level of PO and to explore their validity.

**Materials and methods:**

A clinical sample of 118 patients was recruited from a 4-weeks in-patient cognitive psychotherapy program. Beside the IPO, Structured Clinical Interview for the fourth edition of the Diagnostic and Statistical Manual of Mental Disorders, (DSM-IV.) Axis I and II, Symptom Check List-90 (SCL-90), State-Trait Anger Expression Inventory and Dissociative Experience scale (DES). Two types of analyses were conducted: a person-centered (latent profile) analysis and various variable-centered tests to confirm the factor structure of IPO and calculate group differences.

**Results:**

The three-factor (CFI = 0.990, TLI = 0.990, RMSEA = 0.022, SRMR = 0.089) and the five-factor (CFI = 0.995, TLI = 0.995, RMSEA = 0.014, SRMR = 0.090) models of the IPO was supported. Latent class analysis identified three subgroups of PO: “Well-integrated,” “Moderately integrated,” and “Disintegrated” classes. There were no significant differences between the three classes in the number of Axis 1 diagnoses (*p* = 0.354; η^2^ = 0.01). Group differences in the number of PDs, the number of PD symptoms as well as in the presence of borderline and depressive PD were significant (all *p* < 0.001; *V* = 0.35–0.42; η^2^ = 0.15–0.26). Persons with more severe PO problem level had higher rates of psychopathological symptoms, state and trait anger, and dissociative characteristics (all *p* < 0.001; η^2^ = 0.13–0.36).

**Conclusion:**

The IPO can be an appropriate instrument to measure the severity of personality disorganization and to classify participants along a continuum of severity in this regard. Our results present further evidence that the severity of personality dysfunction, the central dimension of the ICD-11 and the Alternative Model for PDs is detectable with an instrument, the IPO, that was initially developed to detect the disturbances in PO.

## Introduction

The categorical nature of personality disorders (PDs) classifications in the fourth edition of the Diagnostic and Statistical Manual of Mental Disorders (DSM-IV) and the tenth revision of the International Classification of Diseases (ICD-10) (1, 2) did not provide sufficient flexibility to administer clinically important information about the severity and nature of personality disturbance. The severity of personality disturbance received a more central role in the rationale for the reclassification of PD in the ICD-11 where the committee proposed “to make the primary classification based on the severity of personality disturbance” [(3–5), p. 246]. The proposal included classifying the severity of personality variation into five levels: (1) No PD, (2) Personality difficulty, (3) Mild PD, (4) Moderate PD, and (5) Severe PD.

Furthermore, the differentia specifica of PDs is defined as “a pattern of general impairment in human relationships that prevents mutual understanding” in the ICD-11 [(5), p. 250], thus impairment of interpersonal functioning. Thus, the severity of a PD is defined by the pervasiveness and complexity of interpersonal disturbance. A review of previous attempts to define PD in terms of severity (6) concluded that the more domains of cognition, affectivity, control over impulses, the gratification of needs, and interpersonal relationships are impaired, the more severe the PD is.

The fifth edition of the Diagnostic and Statistical Manual of Mental Disorders (DSM-5) attempts to solve this problem by introducing the levels of personality functioning (LPF) scale, which was proposed for further research in “Alternative DSM-5 Model for Personality Disorders (AMPD)” in Section III of DSM-5. LPF is evaluated on a continuum of self-level disturbances (identity and self-direction) and interpersonal disturbances (empathy and intimacy) and rated one (little or no impairment) to five (severe impairment) (7). The DSM claims that “impairment in personality functioning predicts the presence of a PD, and the severity of impairment predicts whether an individual has more than one PD or one of the more typically severe PDs” (p. 762). Both the presence and higher degrees of impairment result in poorer functioning compared to having only one or a less severe form of PD. Besides severity ratings, ICD-11 and DSM-5 introduced trait domain qualifiers for PD: negative affectivity, detachment, disinhibition, dissociality/antagonism (ICD-11/DSM-5), anankastia (ICD-11), and psychoticism (DSM-5) (8, 9).

In addition, there are other measuring instruments that have been developed to assess personality impairment. E.g., the Operationalized Psychodynamic Diagnostics Structure Questionnaire (OPD-SQ) is developed from the fourth, structural axis of the OPD system, (10, 11).

Based on the descriptions of PD severity in the ICD-11 and DSM-5, the need emerges for tools to separate groups of people with different severity of personality dysfunction based on personality disturbances. Bach and Simonsen (12) propose that the ICD-11 classification of PD severity and the DSM-5 Levels of Personality Functioning Scale (LPFS) are essentially comparable to Kernberg’s Level of Personality Organization (PO) approach. Kernberg’s model of PO is an example of the model’s development based on the level of personality functioning (13). The model published by Kernberg et al. (14) is based on the levels of PO. This was one of the first attempts to categorize personality pathology according to severity. The model includes three levels of POs: (1) the psychotic (PPO), (2) the borderline (BPO), and (3) the neurotic personality organization (NPO). Kernberg delineated the level of PO based on the following key aspects of intrapsychic functioning: (a) use of psychological defense mechanisms, (b) extent of reality testing, (c) the level of identity integration, (d) the control of aggression, and (e) moral functioning (ethical behavior, ideals, and values) (15, 16). According to this model, those with (a) more frequent use of primitive defense mechanisms such as splitting and projective identification and less frequent use of more mature defense mechanisms such as repression and rationalization to cope with external and internal stressors and conflicts, (b) incapacity to differentiate self from non-self, to distinguish intrapsychic from external sources of stimuli, and to maintain empathy with ordinary social criteria of reality, (c) poorly defined sense of self and low level of self-other differentiation and identity, (d) inability to control aggression, and (e) contradictory and incompletely internalized value system is considered to have a more severe PD. According to Kernberg, a lower level of PO is associated with more severe interpersonal dysfunction (17), more first [Berghuis et al. (18)] and second axis symptoms, more severe PD types (14), more severe dissociative symptoms (19), anger, and aggressive dyscontrol (17, 20).

Several rating scales and self-reported questionnaires were developed to measure the level of PO and to validate the model empirically. Kernberg and his colleagues (21, 22) constructed the self-report questionnaire called Inventory of Personality Organization (IPO) and a related semi-structured interview. Diguer and Normandin (23) developed the Personality Organization Diagnostic Form-I (PODF) that was further improved by Gamache et al. (24) and named the PODF-II. The original version of the (IPO-3) assesses identity diffusion, primitive defenses, and reality testing (16, 20, 25, 26). Later two scales were added, aggression and moral values (IPO-5). Studies have supported the reliability and validity of the IPO-3 (20, 25) and IPO-5 (18, 27). The construct validity of IPO was confirmed by the study of Smits et al. (28), which found that IPO was able to differentiate normal controls from patients suffering from non-personality pathology and PDs.

The aim of the current study was to identify severity groups according to the level of PO and to explore their validity. More specifically, our hypotheses were that PO problem severity is positively associated with the number of (1) Non-personality pathology diagnoses, (2) non-personality pathology symptoms (SCL-90), (3) PD symptoms, (4) PD diagnoses, (5) PD Cluster A and B diagnosis, (6) dissociative symptoms, and (7) higher levels of anger (state and trait anger, anger expression, lower level of anger suppression, control of anger expression, and calming down of angry feelings).

## Materials and methods

### Data collection

Patients were recruited from a 4-weeks cognitive psychotherapy program in the Department of Psychiatry and Psychotherapy at Semmelweis University, Budapest, Hungary prior to receiving the intervention. Participation in the study was voluntary, with no incentives offered. Exclusion criteria were operationalized by following the standard practice of PD research of excluding subjects whose non-personality pathology state may interfere with an assessment of their more enduring personality traits or symptoms (29). Participants meeting the criteria of current or past diagnosis of the organic mental syndrome, CNS neurological disease, and those having schizophrenia, schizoaffective disorder, or other psychotic disorders, current hypomanic/manic episode, or substance withdrawal syndrome were excluded from the study, as well as those who did not have the mental competency and ability to complete the self-report questionnaires or provide informed consent. Prior experience with psychotherapy was not an exclusion criterion. To enhance the generalizability of the results, no other exclusion criteria were used.

All participants gave written informed consent to participate in the study prior to assessment and agreed to use their anonymous data for research purposes. The design was approved by the “institute”s research ethics committee.

### Participants

Overall, 188 patients participated in the study. One participant was under 18 years old (18), therefore this data was excluded from the study. Further 6 “participants” data did not contain any values on the IPO, thus these were excluded as well. This left 181 “participants” data for the current study.

### Structured interviews for clinical assessment

Non-personality pathology diagnoses were assessed by the Hungarian version of the Structured Clinical Interview for DSM-IV Axis I disorders [SCID I, (30, 31)]. Assessment was carried out by trained and experienced psychiatrists or clinical psychologists. Diagnoses were classified into the following main categories: Anxiety Disorders (Generalized Anxiety Disorder, Panic Disorder Without Agoraphobia, Panic Disorder With Agoraphobia, Agoraphobia Without History of Panic Disorder, Post-traumatic Stress Disorder, Specific Phobia, Social Phobia, Anxiety Disorder NOS), Obsessive Disorder (Obsessive-Compulsive Disorder), Depressive Disorders (Dysthymic Disorder, Major Depressive Disorder: Single Episode, Recurrent, Depressive Disorder NOS), Bipolar Disorders (depressive episode, Bipolar I Disorder, Bipolar II Disorder, Cyclothymic Disorder, Bipolar Disorder NOS), Somatoform Disorders (Conversion Disorder, Pain Disorder, Somatization Disorder), Dissociative Disorder (Depersonalization Disorder, Dissociative Fugue, Dissociative Disorder NOS), Substance-Related Disorders (Substance use disorders: dependence, abuse: alcohol, amphetamine-like, cocaine, cannabis, hallucinogen, sedative, hypnotic, anxiolytic), Impulsive Eating Disorders (Binge eating, Bulimia, and Binge-Purging type Anorexia), Restrictive Anorexia Nervosa. None of the psychotic diagnostic categories were met.

Personality disorder diagnoses were determined by the Hungarian version of the Structured Clinical Interview for DSM-IV Axis II PDs [SCID-II; (32, 33)]. Diagnostic ratings interpreted in two ways: (1) as individual diagnoses based on the interview, (2) counting individual symptoms (out of 127 possible symptoms) where only the symptoms rated as “3” (PD symptom) were considered be met (positive), ratings of 1 and 2 were counted as non-met (negative). The summary score was used as a severity measure. The interview was administered by a trained professional. Assessment was carried out by a trained and experienced psychiatrist or by a clinical psychologist.

### Self-report instruments

The [IPO; (21)] is an 84-items self-report questionnaire. Items are rated on a five-points Likert-type scale ranging from never true to always true. The IPO has three primary scales, including Primitive Psychological Defenses (PPD; 16 items), Identity Diffusion (ID; 21 items), and Reality Testing (RT; 20 items), and two additional scales, Aggression (AGG; 18 items) and Moral Values (MV; 8 items with 2 PPD items and 1 ID item). PPD refers to primitive psychological defenses such as projection, denial, dissociation, externalization, splitting, idealization, and devaluation. ID measures facets related to poorly integrated identity, particularly concepts of self and significant others, as well as inadequate perception and understanding of others. RT covers items related to the “capacity to differentiate self from non-self, to distinguish intrapsychic from external sources of stimuli, and to maintain empathy with ordinary social criteria of reality” [(34), p. 120]. AGG consists of items related to control over aggressive impulses, self-harming behavior and ideations, manipulation of others, and sadistic aggression. MV assesses the psychodynamic construct of superego pathology. Psychometric characteristics of the IPO in our sample suggest that the five scales display adequate internal consistency: PPD (α = 0.80), ID (α = 0.87), RT (α = 0.87), RT (α = 0.90), MV (α = 0.70).

The IPO was translated to Hungarian using two independent translations of the measure: one was carried out by a bilingual (English-Hungarian) person who was unfamiliar with the concept of PO, and the second one by one of the authors (ZU). Translations were discussed, improved, and assembled into a single version. In the next step, a native English-speaking person back translated this version who was not involved in the initial translation. This back-translation was compared to the original IPO and changes to the Hungarian translation were made where necessary.

In order to assess the level of general distress and the severity of nine symptom dimensions, the Hungarian version of the *Symptom Check List-90* [SCL-90-R; (35); R. (36); SCL-90; R. (37)] was used. The SCL-90-R is a 90-items self-report questionnaire covering a wide range of psychopathological symptoms that are rated for severity during the past week. This instrument reliably distinguishes between clinical and normal population (38). Psychometric characteristics of the SCL-90 suggest that the nine scales display adequate internal consistency in the current sample: Somatization α = 0.91, Obsessive-Compulsive Behavior α = 0.87, Hostility α = 0.78, Phobic Anxiety α = 0.85, Depression α = 0.90, Psychoticism α = 0.76, Anxiety α = 0.80, Paranoid Ideation α = 0.80, and Interpersonal Sensitivity (IS) α = 0.86. The Global Severity Index (GSI, α = 0.98) consists of the mean of all items. The Personality Severity Index (PSI α = 0.92) is defined as the mean value of the SCL-90-R subscales IS, Hostility (HOS), and Paranoid Ideation (PAR) and has been found to be strongly related to (severe) personality pathology (39, 40). Finally, the Current Symptom Index (CSI, α = 0.97) is the mean value of the scores on its six subscales: (Somatization, Obsessive-Compulsive Behavior, Depression, Anxiety, Phobic anxiety, Anxiety, and Psychoticism).

The State-Trait Anger Expression Inventory [STAXI-2; (41)] was developed to assess the intensity of anger as an emotional state (State Anger) and the disposition to experience angry feelings as a personality trait (Trait Anger). STAXI-2 is a 57-items questionnaire, and its items are scored on a four-points Likert-type scale for all questions. Psychometric characteristics of the STAXI-2 in our sample suggest that its scales display adequate internal consistency. The State Anger (α = 0.87) scale assesses the intensity of anger as an emotional state at a particular time, whereas the Trait Anger (α = 0.90) evaluates a “person”s general predisposition to become angry. Finally, the Anger Expression and Anger Control scale (α = 0.77) incorporates items regarding the expression of anger-related traits.

The Dissociative Experience Scale [DES; (42, 43)] is a 28-items self-report measure of the frequency of dissociative experiences of varying severity. To answer DES questions subjects circle the percentage of time (given in 10% increments ranging from 0 to 100) that they have the experience described. In a validation study of the Hungarian DES by Kocsis-Bogár (44) found that DES had a three-factor structure. In our sample each scale had adequate internal consistency: Amnesia (α = 0.85), Absorption in imagination (α = 0.85), Depersonalization-Derealization (α = 0.78).

### Data analysis

Two types of analyses were conducted: a person-centered (latent profile) analysis and various variable-centered tests to confirm the factor structure of IPO and calculate group differences.

Before calculating the latent classes, confirmatory factor analysis (CFA) was used to confirm the initial five-factor structure and the three-factors structure of the IPO. We allowed for covariance between factors using Weighted Least Squares (WLSMV) which is robust to item non-normality (45). According to Hooper et al. (46), the Comparative Fit Index (CFI) > 0.95, Parsimony Goodness-of-Fit Index (PGFI) > 0.90 (although this is not a fixed threshold), and Tucker-Lewis Index (TLI) > 0.95 indicate good fit, in addition to the Root Mean Square Residual (RMSE) being lower than 0.06 and the Standardized Root Mean Square Residual (SRMR) being 0.09 or lower.

Mixture modeling was conducted to identify distinct groups of participants using the IPO scales as continuous indicators. A special case of mixture models is latent profile analysis (LPA) (47, 48) which is a method for identifying unmeasured class membership among individuals using continuous observed variables (in this case factor scores of IPO). Several models were tested, estimating fit indices based on 2–5 classes and 3 or 5 IPO factors in the entire sample (*N* = 181).

In order to select the best fitting model using LPA, the Akaike Information Criteria (AIC), the Bayesian Information Criteria (BIC), the Sample Size Adjusted BIC (SSABIC) and entropy was calculated. Lower AIC, BIC, and SSABIC values indicate better model fit and higher entropy indicates better classification quality. While AIC, BIC, and SSABIC values do not have a valid threshold, it was suggested that values around 0.40, 0.60, and 0.80 as representing low, medium, and high entropy. In the final determination of the number of classes, the likelihood-ratio difference test (Lo-Mendell-Rubin Adjusted LRT Test, LMR) was used. This compares the estimated model with a model having one less class than the estimated model (49). A low *p*-value (<0.05) indicates that the model with one less class is rejected in favor of the estimated model.

Following the establishment of the number of latent classes and class membership, group comparison tests were carried out to test the validity of the classes by comparing them along a number of variables to test the proposed hypotheses (i.e., demographic variables, non-personality pathology and PD symptoms, SCL-90, STAXI, and DES). Chi-square tests were used to test the independence of categorical variables, and ANOVA (with “Tukey”s test for *post-hoc* analysis) was used to test class differences on continuous measures.

Mixture modeling (LPA) was conducted in Mplus (49) using the default settings. All other statistical analyses were conducted using R (50) using base packages and psych (51). All data and data analytical scripts are available on the OSF: https://osf.io/f2ag4/.

## Results

### Sample characteristics

Out of the 181 participants, 158 (88.3%) were female. Mean age was 33.6 years (SD: 10.6). In terms of education, 20 completed less than 12 classes, 52 no more than 12 classes, 46 had vocational training, and 52 had a university degree. In 11 cases data on education were missing.

### Latent class analysis

First, the initial, five-factors structure as well as the three-factors structure were tested using CFA. Model fit indices indicated good to excellent fit to the data for both models (three-factors model: χ^2^ = 1666.77, df = 1535, *p* = 0.010, CFI = 0.990, TLI = 0.990, RMSEA = 0.022, SRMR = 0.089, PGFI = 0.861; five-factors model: χ^2^ = 3404.64, df = 3306, *p* = 0.113, CFI = 0.995, TLI = 0.995, RMSEA = 0.014, SRMR = 0.090, PGFI = 0.891).

In the second step, multiple latent class solutions were tested using all five and only the three primary factors. None of the solutions reached local maxima. As depicted in Table 1, AIC, BIC, and SSABIC values decreased as the number of classes increased, although the difference was small. The first non-significant LMR value with the three-factor solution was at four classes, which indicates that the three-class solution was the most appropriate (and the four-class solution is not significantly better). Entropy was still acceptable with this solution. In the case of the latent class models with five factors, the first non-significant LMR value was at three classes, which indicates that the two-class solution was the most appropriate (and the three-class solution is not significantly better). Overall, the three-latent class model based on the three primary factors was chosen as the best-fitting solution for the mixture modeling. The relatively low sample size of the present study might be contributed to having lower levels of statistical power to identify the most appropriate classification solution for the more complex latent models with five factors. In line with that, it was assumed that it might be possible to identify a more precise solution for the simpler and parsimonious classification models with only three factors, considering the given sample size. Moreover, it was considered that the most appropriate three-class solution based on the three-factors model provided a theoretically more relevant and distinctive classification. Finally, the secondary factors of AGG and MV showed high-very high correlations with the primary factors of PPD, RT, and ID in the five-factors CFA model (*r* = 0.66–0.89). Thus, these findings indicate that the factors of AGG and MV might only provide limited additional information capacity over the primary IPO factors, which can also support the decision to retain a model based on the more parsimonious three-factor model. Class membership of the three-class model based on the three primary factors was added to the data accordingly. Average class assignment probabilities were 0.92, 0.94, and 0.91, respectively.

**TABLE 1:** Latent class analysis with multiple solutions.

Number of classes	Number of factors	AIC	BIC	SSAIC	Entropy	LMR	LMR p
2	5	1379.60	1430.90	1380.20	0.88	341.60	0.035
2	3	902.30	934.32	902.70	0.79	167.70	0.008
3	5	1258.54	1329.03	1259.35	0.91	129.96	0.155
**3**	**3**	**840.23**	**885.01**	**840.67**	**0.83**	**65.16**	**0.008**
4	5	1196.76	1286.50	1197.80	0.84	71.45	0.380
4	3	820.40	878.10	821.10	0.80	31.68	0.525
5	5	1158.74	1267.70	1160.00	0.87	48.5	0.062
5	3	800.20	870.70	801.00	0.81	26.921	0.053

The final model appears in bold. AIC, Akaike information criteria; BIC, Bayesian information criteria; SSABIC, sample size adjusted BIC; LMR, Lo-Mendell-Rubin adjusted LRT test.

**FIGURE 1:**
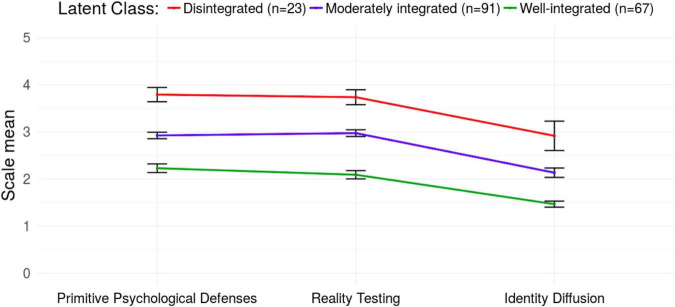
Latent class means on the three primary factors of the Inventory of Personality Organization (IPO) with 95% confidence intervals.

The final class solution is depicted in Figure 1. Latent classes appeared to reflect severity, therefore were labeled as “Well-integrated,” “Moderately integrated,” and “Disintegrated.” In terms of the scale differences, ANOVA indicated significant differences on the IPO primary factors between latent classes (PPD: *F* = 186.28, df = 2, *p* < 0.001, η^2^ = 0.68; RT: *F* = 227.47, df = 2, *p* < 0.001, η^2^ = 0.72; ID: *F* = 96.94, df = 2, *p* < 0.001, η^2^ = 0.52) and on the secondary factors as well (AGG: *F* = 55.90, df = 2, *p* < 0.001, η^2^ = 0.39; MV: *F* = 65.33, df = 2, *p* < 0.001, η^2^ = 0.42).

### Class differences and validity

Comparisons of the latent classes are shown in Table 2. Latent groups did not differ in terms of gender and education, but the Disintegrated group was significantly younger than the other two groups.

**TABLE 2:** Comparison of latent classes.

	Well-integrated (*N* = 67)	Moderately integrated (*N* = 91)	Disintegrated (*N* = 23)	Latent class group difference			
				
				*F*/χ^2^	df	*P*	η^2^/Cramer’s *V*
**Gender *N* (%)**							
Male	10 (15.15%)	10 (11.11%)	1 (4.35%)	χ^2^ = 1.99	2	0.370	*V* = 0.11
Female	56 (84.85%)	80 (88.89%)	22 (95.65%)				
**Education *N* (%)**							
Primary school	3 (4.76%)	4 (4.71%)	3 (13.04%)	χ^2^ = 16.85_a_	10	0.078	*V* = 0.22
Few secondary school classes	1 (1.59%)	7 (8.24%)	2 (8.70%)				
Secondary school completed	20 (31.74%)	21 (24.71%)	11 (47.83%)				
Vocational training	14 (22.22%)	29 (34.12%)	3 (13.04%)				
University degree	24 (38.10%)	24 (28.24%)	4 (17.39%)				
Other	1 (1.59%)	0 (0.00%)	0 (0.00%)				
Age M (SD)	35.62 (11.25)^b^	33.95 (9.98)^b^	27.83 (8.23)^a^	*F* = 4.91	2	0.008	η^2^ = 0.05
**Non-personality pathology diagnoses *N* (%)**							
Anxiety disorders	26 (38.80%)	30 (32.97%)	8 (34.78%)	χ^2^ = 3.98_a_	6	0.680	*V* = 0.10
Obsessive disorder	1 (1.50%)	6 (6.59%)	2 (8.70%)	χ^2^ = 2.90	2	0.235	*V* = 0.13
Depressive disorders	32 (47.76%)	43 (47.25%)	10 (43.48%)	χ^2^ = 1.27_a_	4	0.867	*V* = 0.06
Bipolar disorders	2 (2.99%)	11 (12.09%)	8 (34.78%)	χ^2^ = 22.58_a_	4	<0.001	*V* = 0.25
Somatoform disorders	3 (4.48%)	3 (3.30%)	0 (0.00%)	χ^2^ = 1.07_a_	2	0.585	V = 0.08
Dissociative disorder	0 (0.00%)	1 (1.10%)	0 (0.00%)	χ^2^ = 0.99_a_	2	0.608	*V* = 0.07
Substance-related disorder	6 (8.96%)	12 (13.19%)	4 (17.39%)	χ^2^ = 2.75_a_	4	0.600	*V* = 0.09
Impulsive eating disorders	7 (10.45%)	11 (12.09%)	4 (17.39%)	χ^2^ = 0.77	2	0.679	*V* = 0.07
Restrictive anorexia nervosa	6 (8.96%)	7 (7.69%)	3 (13.04%)	χ^2^ = 0.65	2	0.721	*V* = 0.06
**Number of non-personality pathology diagnoses present *N* (%)**							
0	12 (17.91%)	19 (20.88%)	4 (17.39%)	χ^2^ = 11.90_a_	10	0.292	*V* = 0.18
1	28 (41.79%)	30 (32.97%)	8 (34.78%)				
2	18 (26.87%)	24 (26.37%)	2 (8.70%)				
3	7 (10.45%)	12 (13.19%)	7 (30.43%)				
4	2 (2.99%)	4 (4.40%)	2 (8.70%)				
5	0 (0.00%)	2 (2.20%)	0 (0.00%)				
Number of non-personality pathology diagnoses present M (SD)	1.39 (1.00)^a^	1.54 (1.21)^a^	1.78 (1.31)^a^	*F* = 1.05	2	0.354	η^2^ = 0.01
**Personality disorder diagnoses *N* (%)**							
Avoidant	11 (21.15%)	23 (29.11%)	10 (43.48%)	χ^2^ = 3.92	2	0.141	*V* = 0.16
Dependent	1 (1.92%)	4 (5.06%)	2 (8.70%)	χ^2^ = 1.79	2	0.409	*V* = 0.11
Obsessive-compulsive	10 (19.23%)	26 (32.91%)	4 (17.39%)	χ^2^ = 4.09	2	0.130	*V* = 0.16
Passive-aggressive	6 (11.54%)	13 (16.46%)	6 (26.09%)	χ^2^ = 2.49	2	0.288	*V* = 0.13
Depressive	9 (17.31%)	36 (45.57%)	15 (65.22%)	χ^2^ = 18.37	2	<0.001	*V* = 0.35
Paranoid	5 (9.62%)	22 (27.85%)	5 (21.74%)	χ^2^ = 6.35	2	0.042	*V* = 0.20
Schizotypal	2 (3.85%)	10 (12.82%)	2 (8.70%)	χ^2^ = 3.03	2	0.220	*V* = 0.14
Schizoid	1 (1.96%)	2 (2.53%)	0 (0.00%)	χ^2^ = 0.59_a_	2	0.743	*V* = 0.06
Histrionic	0 (0.00%)	4 (5.06%)	1 (4.35%)	χ^2^ = 2.66_a_	2	0.264	*V* = 0.13
Narcissistic	1 (1.92%)	6 (7.59%)	2 (8.70%)	χ^2^ = 2.23	2	0.327	*V* = 0.12
Borderline	15 (28.85%)	51 (64.56%)	20 (86.96%)	χ^2^ = 26.83	2	<0.001	*V* = 0.42
Antisocial	1 (1.92%)	4 (5.19%)	2 (8.70%)	χ^2^ = 1.91	2	0.385	*V* = 0.11
Cluster A or B personality disorders	32 (47.76%)	70 (76.92%)	21 (91.30%)	χ^2^ = 21.67	2	<0.001	*V* = 0.35
Cluster C personality disorders	19 (28.36%)	40 (43.96%)	13 (56.52%)	χ^2^ = 7.00	2	0.030	*V* = 0.20
**Number of personality disorder diagnoses present *N* (%)**							
0	22 (32.84%)	8 (8.79%)	1 (4.35%)	χ^2^ = 48.41_a_	16	<0.001	*V* = 0.37
1	31 (46.27%)	33 (36.26%)	2 (8.70%)				
2	6 (8.96%)	14 (15.38%)	7 (30.43%)				
3	2 (2.99%)	13 (14.29%)	5 (21.74%)				
4	5 (7.46%)	10 (10.99%)	4 (17.39%)				
5	0 (0.00%)	8 (8.79%)	2 (8.70%)				
6	0 (0.00%)	3 (3.30%)	2 (8.70%)				
7	0 (0.00%)	1 (1.10%)	0 (0.00%)				
8	1 (1.49%)	1 (1.10%)	0 (0.00%)				
Number of personality disorder diagnoses present M (SD)	1.15 (1.40)^a^	2.34 (1.79)^b^	3.00 (1.57)^b^	*F* = 15.50	2	<0.001	η^2^ = 0.15
SCID-II personality disorder symptoms M (SD)	13.47 (9.63)^a^	25.56 (13.78)^b^	34.26 (11.86)^c^	*F* = 32.06	2	<0.001	η^2^ = 0.26
**SCL-90-R psychopathological symptoms M (SD)**							
Somatization	1.24 (0.91)^a^	1.99 (1.11)^b^	2.26 (0.97)^b^	*F* = 13.65	2	<0.001	η^2^ = 0.13
Obsessive-compulsive behavior	1.30 (0.79)^a^	2.31 (0.86)^b^	2.88 (0.78)^c^	*F* = 43.27	2	<0.001	η^2^ = 0.33
Hostility	0.81 (0.76)^a^	1.55 (0.95)^b^	2.17 (0.92)^c^	*F* = 25.00	2	<0.001	η^2^ = 0.22
Phobic anxiety	1.13 (1.01)^a^	1.91 (1.12)^b^	2.17 (1.01)^b^	*F* = 13.61	2	<0.001	η^2^ = 0.13
Depression	2.00 (0.98)^a^	2.73 (0.93)^b^	3.23 (0.68)^b^	*F* = 19.77	2	<0.001	η^2^ = 0.18
Psychoticism	0.62 (0.49)^a^	1.35 (0.70)^b^	1.84 (0.66)^c^	*F* = 43.21	2	<0.001	η^2^ = 0.33
Anxiety	1.66 (0.89)^a^	2.37 (0.88)^b^	2.95 (0.84)^c^	*F* = 22.87	2	<0.001	η^2^ = 0.20
Paranoia	0.79 (0.71)^a^	1.80 (0.90)^b^	2.37 (0.98)^c^	*F* = 41.68	2	<0.001	η^2^ = 0.32
Interpersonal sensitivity	1.19 (0.82)^a^	2.14 (0.95)^b^	2.89 (0.77)^c^	*F* = 39.31	2	<0.001	η^2^ = 0.31
Personality severity index	0.98 (0.64)^a^	1.89 (0.81)^b^	2.55 (0.74)^c^	*F* = 49.24	2	<0.001	η^2^ = 0.36
Current symptom index	1.36 (0.69)^a^	2.14 (0.80)^b^	2.60 (0.67)^c^	*F* = 32.64	2	<0.001	η^2^ = 0.27
Global severity index	1.27 (0.63)^a^	2.07 (0.74)^b^	2.58 (0.65)^c^	*F* = 40.38	2	<0.001	η^2^ = 0.31
**STAXI-2 state-trait anger expression M (SD)**							
State anger	1.23 (0.39)^a^	1.45 (0.60)^b^	1.95 (0.90)^c^	*F* = 13.55	2	<0.001	η^2^ = 0.13
Trait anger	1.12 (0.59)^a^	2.62 (0.64)^b^	3.14 (0.76)^c^	*F* = 24.86	2	<0.001	η^2^ = 0.22
Anger expression and anger control	2.49 (0.68)^a^	2.38 (0.60)^a^	2.51 (0.80)^a^	*F* = 0.47	2	0.479	η^2^ = 0.01
**DES dissociative experiences M (SD)**							
Amnesia	3.49 (7.37)^a^	11.74 (13.61)^b^	21.66 (17.64)^c^	*F* = 20.49	2	<0.001	η^2^ = 0.19
Absorption in imagination	12.27 (8.95)^a^	27.29 (17.37)^b^	45.65 (20.54)^c^	*F* = 44.77	2	<0.001	η^2^ = 0.33
Depersonalization-derealization	2.11 (3.94)^a^	14.07 (16.17)^b^	25.43 (17.60)^c^	*F* = 31.01	2	<0.001	η^2^ = 0.26

Letter “a” in subscript related to Chi-square statistics indicate that Chi-square approximation is only indicative due to low cell count. Means in the same row that do not share the same letters in subscripts are differ at *p* < 0.05 level.

The majority of the participants (81%) met at least one non-personality pathology disorder. Non-personality pathology diagnostic categories were independent from the latent class membership with the exception of bipolar disorder. Depressive disorders (depression and dysthymia) were the most common in all three groups. However, there were no statistical differences in the number of non-personality pathology diagnoses patients had across groups.

On average, the Well-integrated group had the lowest number of PD diagnoses, followed by the Moderately integrated group, and the Disintegrated group. The Well-integrated group typically had 1 PD, whereas the Disintegrated group typically had at least 2 diagnoses. Group difference in the number of PDs present was significant (Table 2).

The presence of borderline PD and depressive PD (and to a lesser extent paranoid PD) was strongly associated with latent class membership. Table 2 reveals that the Disintegrated group is characterized mostly by borderline PD and depressive PD. Overall, there were increasing prevalence rates in the classes of Well-integrated, Moderately integrated and Disintegrated, respectively, in terms of having at least one Cluster A or B PD, and having at least one Cluster C disorder. Both the presence of A or B cluster PD, and the presence of a cluster C PD were associated with latent class membership.

In total there were 127 PD symptoms assessed in SCID II. ANOVA revealed significant group differences in the number of symptoms met (coded as 3). The Well-integrated class had the lowest number of symptoms, followed by the Moderately integrated class, and the Disintegrated class – with significant differences in each *post-hoc* comparisons (Table 2).

There were significant differences in the subscales of SCL-90 as well (Table 2). The highest symptomatic levels were shown in the Disintegrated class, followed by the Moderately integrated class and by the Well-integrated group (note: for the subscales of somatization, phobic anxiety and depression there were no statistically significant differences between the Disintegrated and Moderately integrated classes) The highest values were reported on the scale of Depression in all three groups.

State-Trait Anger Expression Inventory scores showed a similar pattern as the SCL-90 subscales. Class differences on the state and trait anger were significant with the Disintegrated group scoring the highest, followed by the Disintegrated and the Moderately integrated classes (Table 2). Non-significant overall class difference was presented in terms of the Anger Expression and Anger Control scale (Table 2).

Statistical calculations confirmed group differences on the subscales of the Dissociative Experiences Scale (DES). In each scale the Disintegrated group presented the highest scores, followed by the Moderately and the Well-integrated classes (Table 2). Although each group scored high on the Absorption in imagination scale, the Disintegrated group scored by far the highest.

## Discussion

The aim of the current study was to explore whether severity groups according to the problem level of PO can be identified, and whether these severity groups can be validated by other symptomatology. More specifically, our hypotheses were that a more severe PO problem level is associated with (i) more Axis-I diagnoses, (ii) more PD symptoms, (iii) more PD diagnoses, (iv) more Cluster A and B diagnoses, (v) more severe symptoms measured by SCL-90, (vi) more severe dissociative symptoms measured by DES, and (vii) higher level of state and trait anger, higher anger expression, lower level of anger suppression and control of anger expression, and calming down of angry feelings.

First, we identified three levels of severity of latent class of PO: disintegrated, moderately integrated, and well-integrated. Bach and Simonsen (12) compares the level of severity measured by the ICD-11 severity of personality disturbances, Kernberg’s levels of PO, and LPF of DSM-5 AMPD modell. Based on this comparison our three levels of severity fit well with the ICD-11 mild, moderate and sever level of personality disturbances.

In contrast to our expectations, results showed no significant differences between the three classes along the non-personality pathologies, as our first hypothesis suggested. This is in line with the results of a separate study that examined different forms of anxiety from the point of self-organization. Results showed that the distribution of different anxiety disorders of non-personality pathology is similar on all levels of self-organization (52). Fischer-Kern and her colleagues (53) examined the co-occurrence of Axis I and Axis II diagnoses with the severity of PO impairment and found a significant relationship when they examined lifetime occurrences. However, only some of the STIPO dimensions corresponded with the number of current non-personality pathology diagnoses. The mentalizing capacity, another structural aspect of personality functioning, did also not correspond with the number of current non-personality pathology diagnoses (53). The reason behind this phenomenon could be that patients with more impaired PO get several different non-personality pathology diagnoses in their lifetime, but not more than a patient with neurotic PO.

The results supported the second and third hypotheses. Our findings correspond with former results regarding the connection between lower levels of PO and PD traits (53). Di Pierro and her colleagues (54) also found that the severity of the PD symptoms measured with SCID II are in correlation with the impairment in personality functioning, measured with a 12-items version of the Level of Personality Functioning Scale. Clark and her colleagues (55) review studies that have used the measures of personality impairment as conceptualized in ICD-11 or using the AMPD and found that PD comorbidity and personality impairment correlate. There is also evidence that psychiatric symptoms and interpersonal and intrapersonal problems are in a significant and positive relation with how many criteria of the 11 PDs of the DSM-III-R the person met (56).

Our fourth hypothesis is also supported by our data. Previous research shows similar results: and Hörz et al. (57) found that PO impairment correlates with cluster A and even higher with B diagnoses. The research of Bender and his colleagues (58) also supports our findings: they found that patients with schizotypal and borderline PDs have more contact with the mental health system, get more likely to receive antipsychotic medical treatment, and get more often hospitalized than avoidant and obsessive-compulsive PDs. This is consistent with Kernberg’s theory about the severity of PO impairment – PDs in cluster A and B are associated with aggression and dyscontrol and primitive defenses, while PDs in cluster C includes disorders characterized by less aggressive, more anxious quality, which is a more mature personality functioning (14, 26). Regarding the relationship between PO and borderline PD, it should be emphasized that PO also showed a relationship with bipolar disorder. It might be possible that the positive link between more severe personality disorganization with borderline PD and bipolar disorder is explained by common underlying characteristics, such as mood instability, emotion dysregulation, and impulsivity.

As a summary of these findings, it can be stated that the more disorganized a personality structure is, the more PD symptoms and a number of comorbid PDs are present, especially from Clusters A and B. However, the PO is unrelated to non-PD symptoms. These results are in line with the results reviewed by Crawford and his colleagues (6), which showed a connection between PD severity and the impairment of the domains of cognition, affectivity, control over impulses, gratification of needs and interpersonal relationships. In addition, we refer to the particularities of the ICD and DSM system: The ICD and DSM diagnostic system was not designed until late to isolate two patients diagnosed with identical PDs based on the severity of their state. The ICD-11’s severity of personality dysfunction addresses this problem (3). The AMPD system introduced by DSM-V is a first attempt at switching toward a dimensional approach of PD-s, making dimensional evaluation at least possible The IPO questionnaire is suitable to distinguish these patients, and the obtained level of severity is also in accordance with those listed in the DSM system.

Our fifth hypothesis was also supported. Previous research supports our findings: Preti and his colleagues (59) found a weak connection between SCL-90 scores and the IPO, but the association was stronger with the subscales related to BPD features causing behavioral problems: impulsivity and anger. This is in line with Kernberg’s theory that aggressive strivings are an important factor in the maintenance of identity-diffusion, or, in certain cases they even dominate the early development so much that it leads to borderline PO (60, 61).

Data support our sixth hypothesis: persons categorized with more severe PO level score higher on the DES. Kernberg comprehends dissociation as a product of primitive defense mechanisms e.g., splitting, and is related to severe PO states such as borderline PO and psychotic PO (61). Spitzer et al. (19) conducted the only research using DES and a PO measuring instrument to our knowledge. Their results contradict ours. They found a positive connection only between the IPO subscales primitive defenses and reality testing vs. the milder aspects of dissociation. The different outcome could be a result of the different admission criteria (e.g., non-clinical subjects and outpatient subjects were included in this study).

Our anger related hypotheses have also been verified. According to the theory of Kernberg, patients with severe PDs have a higher level of aggressivity, caused by an inborn disposition and experienced severe childhood trauma (14, 62). Lenzenweger and his colleagues (20) describe in their validation study a connection between PO level and aggressive dyscontrol. Critchfield found that lower levels of PO are results in more severe states of anger and hostility and more pronounced relational anxiety (17). Tweed and Dutton (63) found in their research about batterers a significant relationship between PO level and physical aggression.

### Theoretical considerations

The results of our study indicate that the IPO questionnaire offers an effective diagnostic and symptomatic differentiation between the various severity levels of patient-population. According to the model of Otto Kernberg, this should be imagined as a continuum, and the particular diagnostic categories can be placed alongside this continuum, with occasional overlaps. An important question that occurs is to what extent can the different PDs be conceived as standalone categories. Following Kernberg’s theory the distinct symptomatic profiles of the PDs are specified by the differences between temperament and the defense mechanisms, and by certain structural components (such as the quality of the super-self-structure), however, without exception the operational aspects also measured by the IPO, the identity-diffusion, the primitive defenses, and the reality testing are the ones to be found in the background (64). We must point out, that Kernberg’s model does not include the assessment of personality traits (criterion B in ICD-11), this must be done additionally to IPO. In cases of lower operational standards of these later aspects a more severe symptomatic profile and a larger number of more severe diagnoses are to be expected. According to this model, beyond the non-personality pathology and PD diagnoses, their underlying factors are also informative (A further question that leads beyond the limits of this research is the relationship between PDs and the non-personality pathology diagnoses).

### Clinical implications

In clinical practice, the severity of PD patients’ state is often assessed by measuring tools that evaluate the severity of disease groups labeled as non-personality pathology before the implementation of DSM V (e.g., depression questionnaires, SCL-90 questionnaires, etc.). However, the question remains whether the state of the patient is to be evaluated from a semiological, a clinical or a developmental, psychiatric point of view. The IPO is a measuring tool based on such a developmental, psychiatric concept (focused on the development of the self-structure) whose measurement results correlate with those of the semiological measurement tools. Our results prove that the severity of PDs can be accurately assessed based on the measurements of certain personality traits, dividing the patients into distinct categories. The advantage of such categorization as opposed to a purely semiological diagnosis is that it appears to be relatively unchanged by time, thus indicating the severity of the underlying problem even in the symptomatically compensated state of the patient suffering from PD. The further relevance of our research results is that if by measuring these three aspects (identity diffusion, primitive defenses, and reality testing) the severity of the patient’s psychological disorder can be effectively evaluated, then the clinical expert treating PDs can develop precisely these operational aspects of the personality instead of treating the divergent symptoms. Bach and Simonsen (12) provided detailed advice for tentative general and specific psychotherapeutic methods’ treatment strategies for mild, moderate, and severe ICD-11 PD severity, which severity levels are similar to our well-integrated, moderately integrated, and disintegrated severity levels. The measurement of the severity of personality impairment alone does not provide enough information to establish an appropriate psychotherapy treatment. Assessing the trait domain qualifiers is essential to set the treatment foci and the treatment alliance, and altogether: to offer the most expedient treatment-method and style (8).

### Limitations and future directions

There were no psychotic patients involved in the study, which limits the generalizability of the results. Furthermore, there is evidence in the literature that the presence of one PD increases the likelihood of another PD, thus, future studies should account for the effect of multiple diagnoses. Future studies should validate cut-off values to identify well-integrated, moderately integrated, and disintegrated personality structures. Further on, men are underrepresented in the sample, therefore it would be important to include them in future studies. The convenience sampling also contributed to relatively lower prevalence rate of non-personality pathology diagnosis in a psychiatric sample (81%), which might also limit the generalizability of the findings. The disintegrated group is relatively small, and their members are significantly younger than the members of the other two groups. This may restrain the generalizability of our results. Our research has not included the healthy, sine morbo population, nor psychotic persons, therefore it would be beneficial to repeat the research with the inclusion of these segments of the population. Cross-sectional nature of the study did not allow to test causal relationships between the variables. We did not collect data on previous psychiatric and psychotherapeutic treatment which may differentially moderate the severity of non-personality pathology and the PDs.

## Conclusion

In the current study we provided supporting evidence in favor of the theoretical model of personality pathology proposed by Otto Kernberg. We found that the (qualitative) structure of the personality is related to the (quantitative) severity of personality- and non-personality-related symptoms, including dissociative experiences, and anger. Our findings are robust in terms of the source of information: the results point to the same direction regardless of whether they were assessed by a trained clinician or self-reported by the patient.

## Data availability statement

The raw data supporting the conclusions of this article will be made available by the authors, without undue reservation.

## Ethics statement

The studies involving human participants were reviewed and approved by the Semmelweis University’s Research Ethics Committee. The patients/participants provided their written informed consent to participate in this study.

## Author contributions

ZU, ZD, and AM designed the study and supervised the data collection. AM analyzed the data. All authors participated in drafting and writing the manuscript, contributed to its revision, take responsibility for the integrity of the data, and approved the final version of the manuscript.
